# Cytochrome P450 Genes *CYP9A58* and *CYP9A59* Contribute to Pyrethroid Tolerance in *Spodoptera frugiperda*

**DOI:** 10.3390/insects17080836

**Published:** 2026-08-12

**Authors:** Dongliang Su, Weifang Yang, Yanan Fan, Xiuxia Li, Yun Huang

**Affiliations:** Key Laboratory of Agri-products Quality and Biosafety (Ministry of Education), Anhui Province Key Laboratory of Crop Integrated Pest Management, School of Plant Protection, Anhui Agricultural University, Hefei 230036, China; 18515292991@163.com (D.S.); 15623017309@163.com (W.Y.); 19861379116@163.com (Y.F.); lixx@ahau.edu.cn (X.L.)

**Keywords:** *Spodoptera frugiperda*, pyrethroid insecticides, P450 expression, RNAi

## Abstract

*Spodoptera frugiperda* (J.E. Smith) is a notoriously destructive and invasive agricultural pest, causing substantial economic losses worldwide. Research has shown that cytochrome P450s play an important role in detoxifying insecticides. However, the precise molecular mechanism of P450-mediated detoxification of pyrethroids in *S. frugiperda* remains largely unknown. Enzyme activity assays demonstrated the involvement of P450 monooxygenases in pyrethroid detoxification in *S. frugiperda*. Subsequently, the results of induction experiments revealed that *CYP9A58* and *CYP9A59* were significantly upregulated by bifenthrin (up to 4.59- and 5.54-fold) and lambda-cyhalothrin (up to 4.17- and 3.28-fold). Stage- and tissue-specific expression analyses suggested that *CYP9A59* exhibited predominant expression in sixth-instar larvae, and *CYP9A58* was primarily expressed in the moth stage, with both P450 genes showing high transcript levels in the midgut. The functional role of P450s was confirmed by RNAi, where knockdown of either *CYP9A58* or *CYP9A59* significantly increased larval susceptibility to lambda-cyhalothrin, raising mortality by 45.71% and 57.68%, respectively. Furthermore, structural modeling demonstrated the capacity of both P450 enzymes—CYP9A58 and CYP9A59—to form stable complexes with pyrethroid insecticides. Collectively, these results confirm that *CYP9A58* and *CYP9A59* are key mediators of pyrethroid detoxification, conferring insecticide tolerance in *S. frugiperda*.

## Simple Summary

Cytochrome P450s are crucial for insecticide detoxification, yet the molecular mechanism underlying P450-mediated pyrethroid detoxification in *S. frugiperda* remains largely unclear. Enzyme activity assays suggested the involvement of P450s in pyrethroid detoxification, with *CYP9A58* and *CYP9A59* being significantly induced by bifenthrin and lambda-cyhalothrin. RNAi-mediated knockdown of these genes significantly increased larval mortality upon lambda-cyhalothrin exposure. Homology modeling and molecular docking revealed that CYP9A58 and CYP9A59 can form stable complexes with both insecticides, showing notably stronger binding affinity for lambda-cyhalothrin. These findings enhance our understanding of *P450* genes in insecticide stress responses and offer valuable insights into *S. frugiperda* control.

## 1. Introduction

The fall armyworm (FAW), *Spodoptera frugiperda* (J.E. Smith) (Lepidoptera: Noctuidae), is a highly destructive pest native to tropical and subtropical America [1]. Its strong migratory capacity, broad host range, and high fecundity have facilitated its rapid global spread [2,3]. FAW was first detected in Yunnan Province, China, on 11 December 2018 [4], and has since caused substantial crop losses in Yunnan and other Chinese provinces [5,6].

Insecticides remain the primary tool for global control of *S. frugiperda* [7,8]. However, their intensive and prolonged use has driven the evolution of insecticide resistance [9]. Pyrethroids, widely used against lepidopteran pests including *S. frugiperda*, are no exception, with resistance reported in numerous field strains. Field populations from China (Yunnan, Hainan, Tibet, Fujian) collected in 2019 showed moderate to high resistance (up to 317-fold) to lambda-cyhalothrin, fenvalerate, and deltamethrin [10]. Similarly, resistance to various pyrethroids has been documented globally. A Mexican population showed 19-fold resistance to permethrin, while populations from Puerto Rico displayed 48-, 35-, and 25-fold resistance to permethrin, zeta-cypermethrin, and deltamethrin, respectively [11]. Kenyan field populations have also developed resistance to deltamethrin and lambda-cyhalothrin [12].

Insecticide resistance in insects is mainly mediated by target-site insensitivity and metabolic detoxification. In long-term chemically controlled field populations, however, metabolic resistance, driven by the overexpression of detoxifying enzymes, has emerged as the predominant form, while target-site mutations account for a minority of cases. Metabolic resistance is particularly problematic due to the involvement of multiple enzyme families, its broad substrate coverage, and its intrinsic link to cross-resistance. Among these enzyme systems, cytochrome P450s are the most critical, acting as the gatekeepers of phase I detoxification. Many studies have also shown that P450s are important components of insect detoxification pathways, which have evolved to cope with the pressure from harmful substances such as insecticides in the environment [13,14]. In *S. frugiperda*, upregulation of specific P450 genes has been associated with enhanced insecticide tolerance. For example, *CYP321A8* increases deltamethrin tolerance [15,16], whereas *CYP321A9* is involved in the detoxification of emamectin benzoate and cyproflanilide [17]. Similarly, P450 gene upregulation contributing to insecticide detoxification has been reported in other lepidopteran pests, including *Plutella xylostella* [18], *S. litura* [19], and *S. exigua* [20]. However, limited information is available on the functions of P450 genes responsible for pyrethroid detoxification in *S. frugiperda,* and further validation is still needed.

Transcriptomic analyses and literature reports have confirmed that cytochrome P450 monooxygenases, particularly the CYP9A subfamily, play a central role in the metabolic detoxification of insecticides in insects [21,22,23,24]. Among them, *CYP9A58* and *CYP9A59*, as key members of this gene cluster, have not only been demonstrated to participate in resistance development to methoxyfenozide and diamide insecticides but have also been closely associated with resistance to pyrethroids, such as deltamethrin [25,26]. Therefore, we aim to determine whether *CYP9A58* and *CYP9A59* perform similar functional roles in the response of *S. frugiperda* to two pyrethroid insecticides: bifenthrin (Type I, without α-cyano group) and lambda-cyhalothrin (Type II, with α-cyano group). Detoxification enzyme activities following insecticide exposure, as well as the expression profiles of *P450* genes, including their developmental stage-dependent and tissue-specific patterns, were examined. The functional roles of target *P450* genes were validated using RNAi, and their potential interactions with both insecticides were explored through homology modeling and molecular docking. These findings provide insights into P450-mediated detoxification mechanisms and offer valuable implications for FAW management in agricultural crops.

## 2. Materials and Methods

### 2.1. Insect

The *S. frugiperda* laboratory strain, originally collected from maize fields in Wuhan, China (114.80° E, 30.85° N) in 2019, was maintained on an artificial diet without insecticide exposure under standard conditions (27 ± 1 °C, 70 ± 10% RH, 16:8 h L:D). Moths were supplied with a 10% honey solution.

### 2.2. Insecticides

Bifenthrin (technical grade, 99.1% purity) and lambda-cyhalothrin (technical grade, 98.0% purity) were purchased from North Weiye Metrology Technology Institute (Beijing, China) and Jiangsu Yangnong Chemical Group Co., Ltd. (Yangzhou, China), respectively. Acetone (Analytical Reagent, AR) was supplied by Sinopharm Chemical Reagent Co., Ltd. (Beijing, China).

### 2.3. Insecticide Bioassays

The toxicity of bifenthrin and lambda-cyhalothrin to the third-instar larvae of *S. frugiperda* was assessed using the topical application method. Briefly, stock solutions of bifenthrin or lambda-cyhalothrin were prepared in acetone and serially diluted to concentrations of 12.5, 25, 50, 100, 200 and 400 μg/mL for bioassays, respectively. Acetone served as a solvent control. For each concentration, 36 larvae of *S. frugiperda* were tested in three independent replicates. Mortality was recorded 48 h post-application and analyzed using probit analysis.

### 2.4. Detoxification Enzyme Activity Assays

To evaluate the effect of pyrethroid insecticides on the activity of detoxification enzymes in *S. frugiperda*, third-instar larvae were exposed to the LC_25_ of bifenthrin or lambda-cyhalothrin for 24 h. Acetone-treated larvae served as controls. Each treatment included at least three biological replicates, with ten surviving larvae per replicate. Crude enzyme extracts were prepared for subsequent assays of carboxylesterase (CarE), glutathione S-transferase (GST), and cytochrome P450 monooxygenase (P450) activities.

CarE activity was measured using α-NA as a substrate, with fluorescence detection at 595 nm as described previously [27]. GST activity was determined spectrophotometrically at 340 nm for 5 min based on the conjugation of reduced glutathione (GSH) with 1-chloro-2,4-dinitrobenzene (CDNB) [28]. P450 O-deethylation activity was assessed using 7-ethoxycoumarin (7-EC) as the substrate, with fluorimetric detection (excitation 360 nm, emission 460 nm) on a SpectraMax i3x (Molecular Devices, San Jose, CA, USA) [29]. Protein concentrations were determined using the Bradford method [30]. At least three biological replicates were performed for each treatment.

### 2.5. Insecticide Induction

Based on the bioassay results, third-instar larvae of *S. frugiperda* were exposed to the LC_25_ and LC_50_ of bifenthrin or lambda-cyhalothrin for 6, 12, 24, 36, and 48 h to examine pyrethroid-induced effects on P450 gene expression. Each treatment was replicated three times, with thirty larvae per concentration. All surviving larvae were collected and stored at −80 °C for the following experiments.

### 2.6. RNA Isolation, cDNA Synthesis, and Quantitative Real-Time PCR (qRT-PCR)

Total RNA was extracted from *S. frugiperda* larvae using Trizol reagent (Invitrogen, Carlsbad, CA, USA) following the manufacturer’s protocol. Third-instar larvae were used for whole-body induction assays, and sixth-instar larvae were used for tissue-specific expression analyses. RNA quality and integrity were assessed by 1% agarose gel electrophoresis and a NanoDrop spectrophotometer (DeNovix Inc., Wilmington, DE, USA). First-strand cDNA was synthesized from 1 μg of total RNA using HiScript III RT SuperMix for qPCR (+gDNA wiper) (Vazyme, Beijing, China) in a 20 μL reaction volume, according to the manufacturer’s instructions.

qRT-PCR was performed on an ABI Prism 7500 System (Applied Biosystems, Waltham, MA, USA) using Taq Pro Universal SYBR qPCR Master Mix (Vazyme, Beijing, China). Each 20 μL reaction contained 8.2 μL ddH_2_O, 10 μL 2× SYBR qPCR Master Mix, 0.4 μL each of forward and reverse primers, and 1.0 μL cDNA template (300 ng/μL). The thermal cycling conditions were: 95 °C for 30 s, followed by 40 cycles of 95 °C for 10 s and 60 °C for 30 s. Melting curve analysis was performed to verify amplification specificity. Primers were designed using Primer Premier 6.0 (Table 1). For qRT-PCR data normalization, reference genes were selected according to the literature [31] and the coefficient of variation of Ct values from preliminary experiments. Specifically, EF-1α and α-TUB were used to normalize the relative expression levels of target genes in induction and RNAi silencing experiments; SOD and RPL10 were used for developmental stage-specific expression analyses; and RPL10, together with α-TUB, was applied for tissue-specific expression analyses. Relative gene expression levels were calculated using the 2^−ΔΔCt^ method [32]. The raw ΔCt and ΔΔCt values for all gene expression analyses are provided as Supplementary Data (Tables S1–S3). Three biological and three technical replicates were performed for each sample.

### 2.7. dsRNA Synthesis and Microinjection

For dsRNA synthesis, target genes (*CYP9A58* and *CYP9A59*) and a non-targeting negative control gene (GFP) were amplified by PCR using primers incorporating the T7 promoter sequence at the 5′ end (Table 2). The PCR products were purified by LiCl precipitation and used as templates for dsRNA synthesis with the HiScribe^®^ T7 Quick High Yield RNA Synthesis Kit (NEB, Ipswich, MA, USA). Third-instar larvae were injected with 1000 ng of purified dsRNA using a Nanoliter Microinjector (Nanoject III, Drummond Scientific, Broomall, PA, USA). Each treatment comprised three replicates with a total of 150 larvae, and all injected larvae were maintained on an artificial diet.

### 2.8. Insecticide Susceptibility of S. frugiperda After RNAi

At 48 h post-dsRNA injection, surviving larvae were exposed to the LC_50_ of bifenthrin or lambda-cyhalothrin. Mortality was recorded 48 h after insecticide treatment. Each treatment was performed in triplicate, with a total of 36 larvae per treatment.

### 2.9. Homology Modeling and Docking

The three-dimensional structures of bifenthrin (PubChem CID: 6442842) and lambda-cyhalothrin (PubChem CID: 6440557) were retrieved from PubChem (https://pubchem.ncbi.nlm.nih.gov/), accessed on 22 November 2025. Homology models of P450 proteins (CYP9A58 and CYP9A59) were generated using the I-TASSER server (https://zhanggroup.org/I-TASSER/), version 5.1, accessed on 25 November 2025. based on their amino acid sequences. Molecular docking of the insecticides with the P450 models was performed using AutoDock Vina, version 1.2.5., with docking pockets defined according to heme-binding sites identified in the NCBI database and substrate characteristics. Docking results were visualized using PyMOL (http://pymol.org/), version 3.1.0., accessed on 26 November 2025.

### 2.10. Statistical Analysis

All data are presented as the mean ± SE (standard error) from at least three independent biological replicates. Significant differences in the expression level of *P450* genes and enzyme activities were analyzed using one-way ANOVA followed by Tukey’s test. Comparisons between treatment and control groups were performed using Student’s unpaired two-tailed *t*-test. Statistical significance was considered at *p* < 0.05. All statistical analyses were performed and graphs were generated using GraphPad Prism (version 9.0, GraphPad Software, San Diego, CA, USA).

## 3. Results

### 3.1. Effect of Pyrethroid Insecticides on Detoxification Enzyme Activity in S. frugiperda

**Figure 1:**
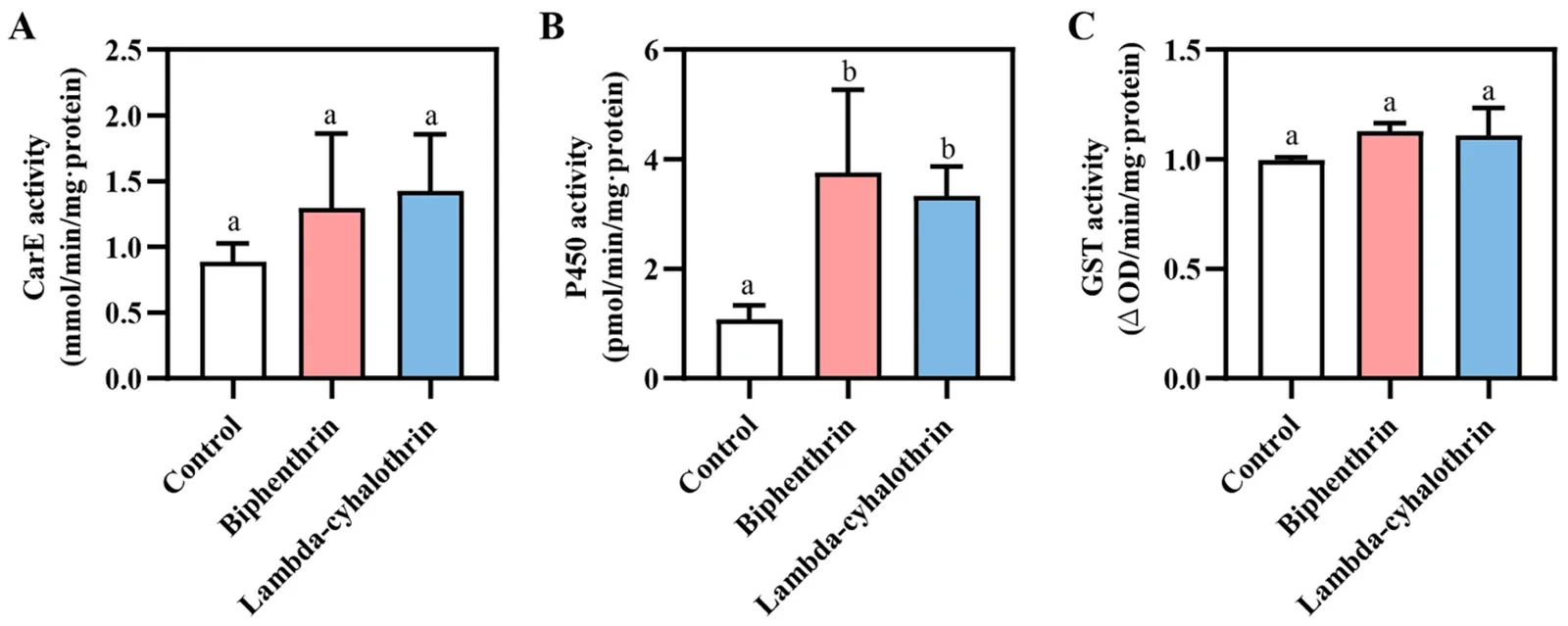
Activities of detoxifying enzymes ((**A**): CarE, (**B**): P450, (**C**): GST) in the third-instar larvae of *S. frugiperda* after exposure to the LC_25_ of bifenthrin and lambda-cyhalothrin, respectively. Enzyme activities marked with different lowercase letters indicate significant differences among insecticide treatments (one-way ANOVA followed by Tukey’s multiple comparison test, *p* < 0.05).

The bioassay results showed that the LC_50_ values of bifenthrin and lambda-cyhalothrin against *S. frugiperda* were 126.585 μg/mL and 88.562 μg/mL, respectively (Table 3). To further assess the role of detoxification enzymes in insecticide response, the activities of CarE, P450, and GST were measured in third-instar *S. frugiperda* larvae exposed to the LC_25_ of bifenthrin and lambda-cyhalothrin for 24 h. The results showed that exposure to bifenthrin and lambda-cyhalothrin significantly increased P450 activity in *S. frugiperda* (3.48- and 3.08-fold, respectively), whereas no significant effect on CarE or GST was observed (Figure 1). This significant induction suggests that P450 plays a vital role in the metabolic defense against these insecticides.

### 3.2. Effect of Pyrethroid Insecticides on the Expression Level of P450 Genes in S. frugiperda

**Figure 2:**
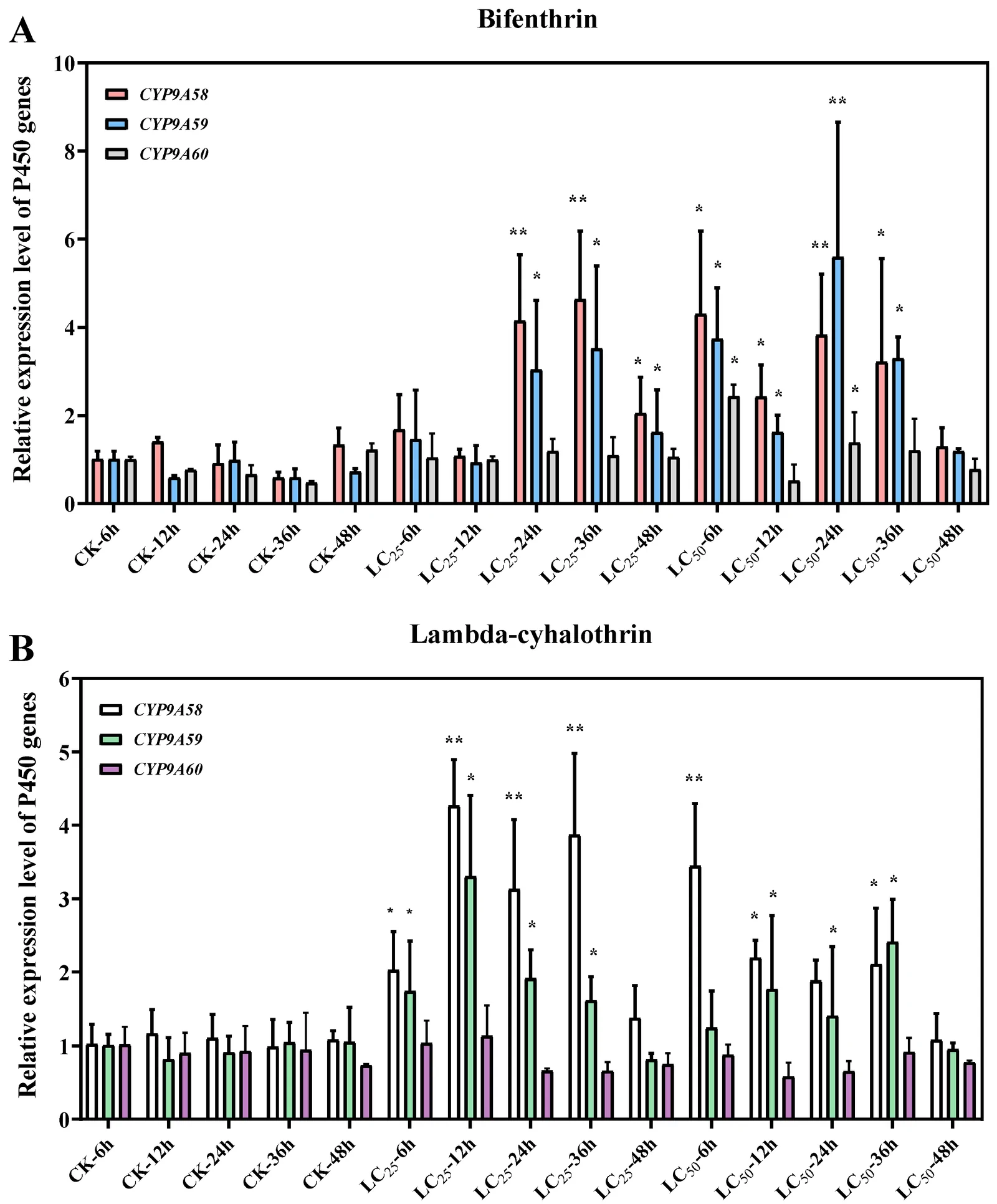
Time-course expression of *CYP9A58*, *CYP9A59*, and *CYP9A60* in third-instar *S. frugiperda* larvae following treatment with sublethal concentrations (LC_25_ and LC_50_) of (**A**) bifenthrin and (**B**) lambda-cyhalothrin. Transcript levels were measured by qRT-PCR at 6, 12, 24, 36, and 48 h post-exposure. Asterisks indicate that there was a significant difference between the treatment and control groups (Student’s unpaired two-tailed *t*-test, * *p* < 0.05, ** *p* < 0.01).

In third-instar larvae of *S. frugiperda*, exposure to the LC_25_ and LC_50_ of either bifenthrin or lambda-cyhalothrin significantly induced the expression of *CYP9A58* and *CYP9A59* at multiple time points. In contrast, the expression level of *CYP9A60* remained largely unchanged, with the exception of a significant response observed at 6 and 24 h post-exposure to the LC_50_ of bifenthrin. Specifically, bifenthrin upregulated *CYP9A58* (up to 4.59-fold) and *CYP9A59* (up to 5.54-fold), while lambda-cyhalothrin increased their expression by 1.84- to 4.17-fold and 1.39- to 3.28-fold, respectively (Figure 2). These differential induction patterns suggest a potential involvement of *CYP9A58* and *CYP9A59* in the stress response of *S. frugiperda* to both pyrethroid insecticides.

### 3.3. Spatiotemporal Expression Patterns of P450 Genes

**Figure 3:**
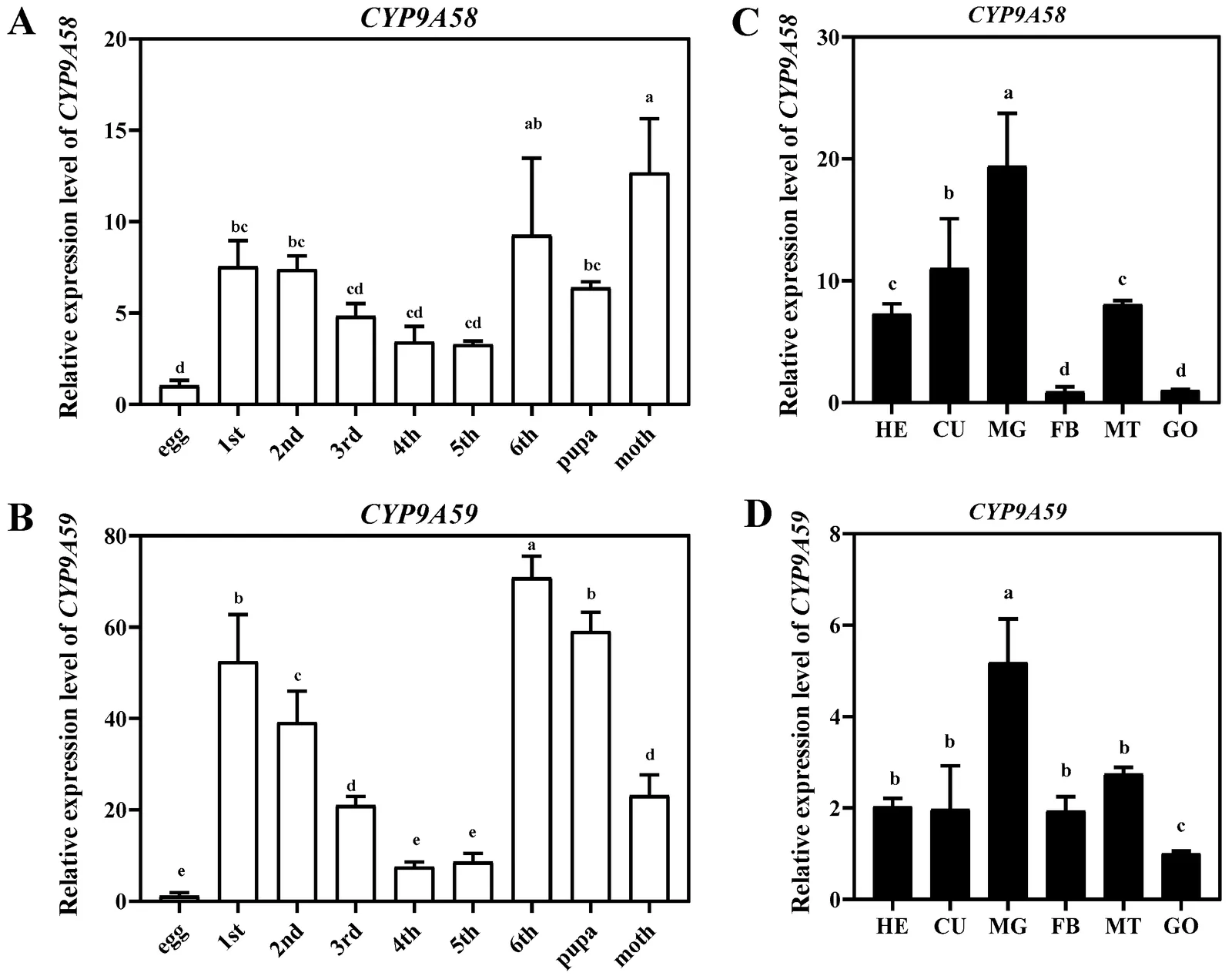
Spatiotemporal expression patterns of CYP9A58 and CYP9A59 in Spodoptera frugiperda. (**A**) Stage-specific expression of *CYP9A58* across different developmental stages (egg, first–sixth-instar larvae, pupa, and moth). (**B**) Stage-specific expression of *CYP9A59* across the same developmental stages. (**C**) Tissue-specific expression of *CYP9A58* in different tissues (head, cuticle, midgut, fat body, Malpighian tubules, and gonads) of sixth-instar larvae. (**D**) Tissue-specific expression of *CYP9A59* in the same larval tissues. Transcript levels were measured by qRT-PCR and normalized to the internal reference gene. Different lowercase letters above the bars indicate significant differences among developmental stages or among tissues for each gene (one-way ANOVA followed by Tukey’s multiple comparison test, *p* < 0.05). Abbreviations: h, hours; qRT-PCR, quantitative real-time polymerase chain reaction; ANOVA, analysis of variance.

The expression of *CYP9A58* and *CYP9A59* genes across nine developmental stages of *S. frugiperda* (egg, first–sixth-instar larvae, pupa, and moth) was further examined. Both P450 genes were highly expressed in first-instar larvae, with levels gradually decreasing through the fifth instar. *CYP9A58* was most abundant in the moth stage (12.30-fold) (Figure 3A), while *CYP9A59* peaked in sixth-instar larvae, reaching 61.44-fold expression relative to the egg stage (Figure 3B). Additionally, the tissue-specific expression profiles of the two P450 genes were examined in the head, cuticle, midgut, fat body, Malpighian tubules, and gonads. The highest expression levels of both *CYP9A58* and *CYP9A59* were detected in the midgut, with increases of 19.34- and 5.18-fold, respectively, compared to the gonads with the lowest expression. Notably, *CYP9A58* and *CYP9A59* were also highly expressed in the head and cuticle, showing 7.27- and 2.03-fold increases in the head and 10.98- and 1.97-fold increases in the cuticle, respectively (Figure 3C,D). These stage-specific and tissue-specific patterns collectively indicate functional divergence among P450 genes in *S. frugiperda*.

### 3.4. RNAi-Mediated Functional Characterization of P450 Genes in S. frugiperda

**Figure 4:**
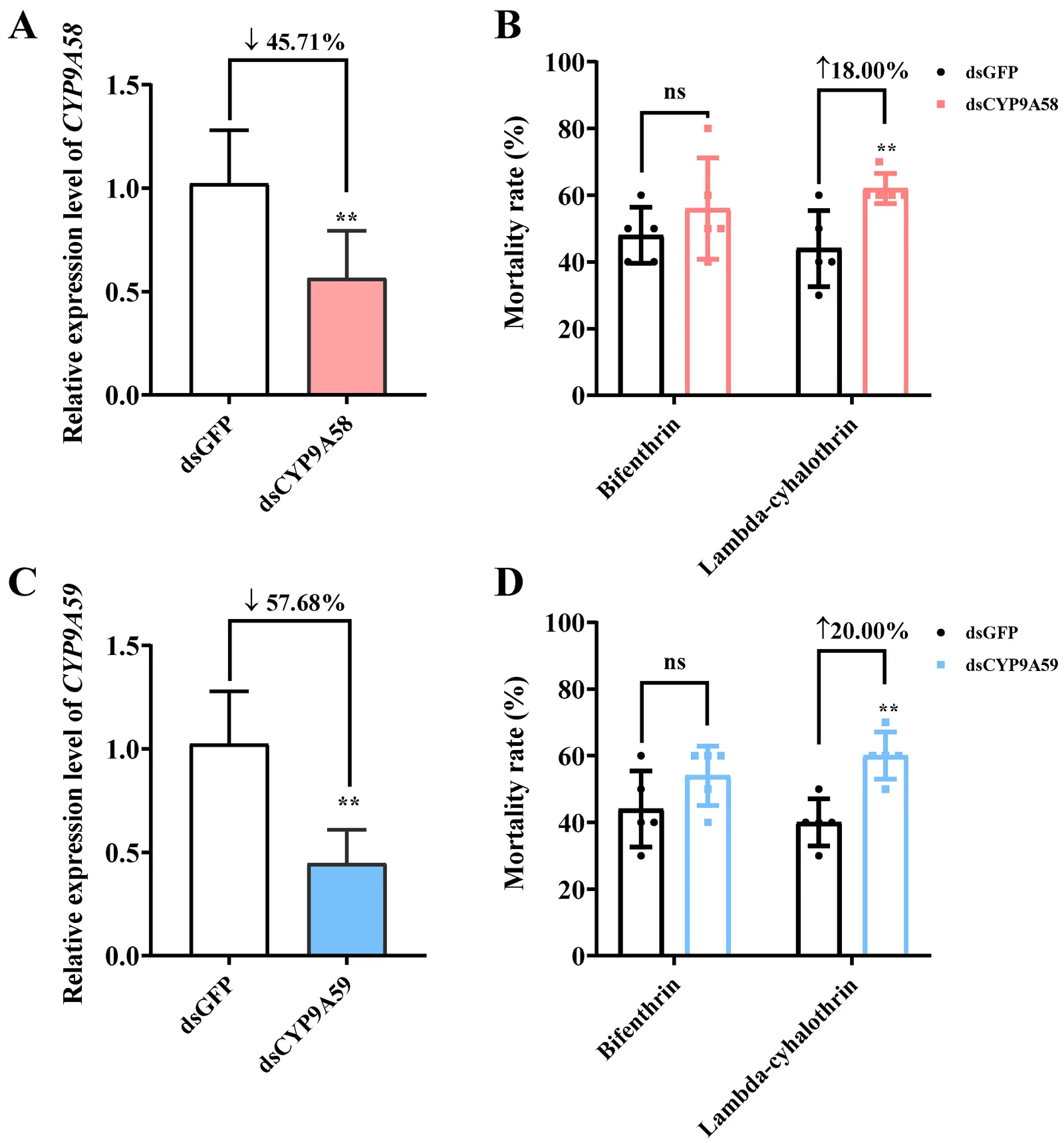
RNAi-mediated functional validation of *CYP9A58* and *CYP9A59* in *S. frugiperda*. (**A**,**B**) Relative expression levels of *CYP9A58* (**A**) and *CYP9A59* (**B**) at 48 h post-injection of dsRNA. (**C**,**D**) Mortality of third-instar larvae exposed to the LC_50_ of bifenthrin or lambda-cyhalothrin at 48 h following silencing of *CYP9A58* (**C**) and *CYP9A59* (**D**). The upward and downward arrows indicate upregulation and downregulation of target gene expression, respectively. Asterisks indicate that there was a significant difference between the treatment and control groups, and ‘ns’ indicates no significant difference (Student’s unpaired two-tailed *t*-test, ** *p* < 0.01).

To validate the role of *CYP9A58* and *CYP9A59* in pyrethroid tolerance, RNAi-mediated knockdown of each gene was performed in *S. frugiperda*. Compared to the dsGFP-injected control, injection of dsCYP9A58 and dsCYP9A59 significantly reduced the expression of the target genes to 45.71% and 57.68% of control levels, respectively (Figure 4A,C). Meanwhile, the mortality rates of *S. frugiperda* exposed to lambda-cyhalothrin increased by 18.00% and 20.00% in the dsCYP9A58 and dsCYP9A59 groups, respectively. In contrast, no significant change in mortality was observed in larvae exposed to bifenthrin (Figure 4B,D). Collectively, these RNAi results support a functional role for *CYP9A58* and *CYP9A59* in lambda-cyhalothrin tolerance but do not provide evidence for their involvement in bifenthrin detoxification, despite transcriptional induction by both insecticides.

### 3.5. Three-Dimensional Structural Modeling and Molecular Docking of P450 Proteins with Pyrethroids

**Figure 5:**
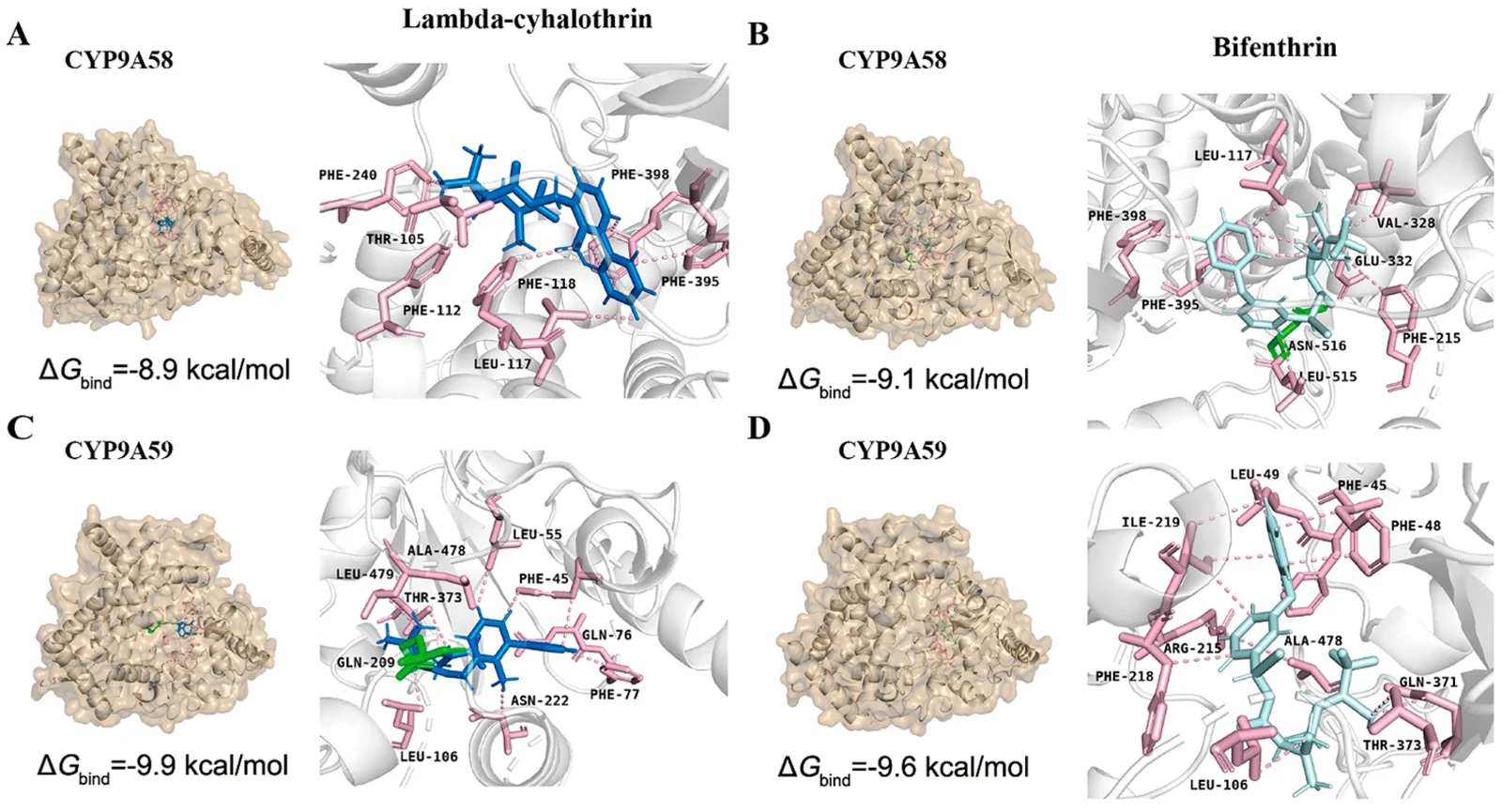
Molecular docking of P450 proteins with pyrethroid insecticides. (**A**,**B**) Predicted binding modes of CYP9A58 with lambda-cyhalothrin (**A**) and bifenthrin (**B**). (**C**,**D**) Predicted binding modes of CYP9A59 with lambda-cyhalothrin (**C**) and bifenthrin (**D**). The ligand-binding pocket is colored dark blue (lambda-cyhalothrin) or light cyan (bifenthrin). Residues are shown in three-letter codes with mature protein numbering.

Homology modeling and molecular docking were used to assess interactions between CYP9A58/CYP9A59 and two pyrethroids. Binding free energy calculations confirmed strong interactions with both insecticides. The CYP9A58- and CYP9A59-lambda-cyhalothrin complexes showed binding energies of −8.9 and −9.9 kcal/mol, respectively (Figure 5A,C), while their bifenthrin complexes exhibited −9.1 and −9.6 kcal/mol, respectively (Figure 5B,D). These results demonstrate that both P450s form stable complexes with each insecticide. Notably, bifenthrin exhibits a stronger theoretical affinity for CYP9A58, whereas lambda-cyhalothrin shows a stronger affinity for CYP9A59.

## 4. Discussion

Cytochrome P450s are key detoxification enzymes in insects that metabolize various endogenous and exogenous compounds, including insecticides, thereby playing a crucial role in the development of insecticide resistance [33,34]. In recent years, the detoxification function of P450s in insects against insecticides has been increasingly reported [35,36,37]. In *S. frugiperda*, *CYP9A58* and *CYP9A59* are induced by methoxyfenozide [38], with *CYP9A58* also contributing to host plant adaptation [39]. Additionally, the synergist PBO has been shown to exert a significant inhibitory effect on cytochrome P450 monooxygenase activity in *S. frugiperda* [40,41,42]. Detoxification enzyme activity analysis in the present study further indicated that cytochrome P450s play a key role in *S. frugiperda*’s detoxification of pyrethroid insecticides, such as bifenthrin and lambda-cyhalothrin. However, whether the *CYP9A58* and *CYP9A59* genes play a similar role in detoxifying pyrethroid insecticides in *S. frugiperda* remains unknown.

Inducibility is considered an important characteristic of *P450* genes, which helps insects to metabolize exogenous compounds more efficiently and thereby enhance their tolerance to insecticides [43,44]. Additionally, the induction of P450s may be closely related to inducers, exposure dose and time [45,46,47,48]. Similar research results were also observed in this study. For example, the expression levels of the *CYP9A58* and *CYP9A59* genes were significantly induced by treatment with the LC_25_ of lambda-cyhalothrin for 12 h, whereas no significant difference was observed following treatment with the LC_25_ of bifenthrin for 12 h. Similarly, no significant difference was found after treatment with the LC_25_ of bifenthrin for 6 h. However, when the concentration was increased, the expression levels of the *CYP9A58* and *CYP9A59* genes were significantly induced by 4.25- and 3.69-fold, respectively, following treatment with the LC_50_ of bifenthrin for 6 h. Furthermore, with prolonged exposure, the expression levels were significantly induced by 4.10- and 3.01-fold, respectively, after treatment with the LC_25_ of bifenthrin for 24 h. It is noteworthy that the upregulation induced by short-term exposure to exogenous insecticides is a transient, non-heritable inducible tolerance that operates only during the exposure period and cannot be transmitted to offspring, which is fundamentally distinct from constitutively inherited resistance in the traditional sense. In the present study, the observed upregulation of *CYP9A58* and *CYP9A59* in our laboratory strain represents an inducible short-term tolerance response, rather than evidence of true resistance evolved in field populations. Therefore, this induced upregulation is insufficient to confer effective protection against the high neurotoxic doses applied at field-relevant label rates, nor can it serve as a basis for judging the occurrence of field resistance.

It should be noted that the LC_50_ values (126.585 and 88.562 μg/mL) obtained in this study are indeed higher than those reported in some previous studies. However, absolute LC_50_ values are highly susceptible to variations in bioassay protocols and operational details, such as solvent type, adjuvant, larval instar, and observation time. Although our laboratory strain has been continuously reared on an artificial diet without exposure to any insecticide selection, it may possess a certain degree of intrinsic tolerance, and its genetic background and rearing history might differ from those of susceptible strains used in other laboratories, all of which could contribute to the observed differences. Nevertheless, the relative mortality trends and the statistically significant differences between treatment groups remain valid under our experimental conditions. Importantly, our subsequent functional assays (RNAi and molecular docking) provide consistent evidence supporting the differential roles of *CYP9A58* and *CYP9A59* in pyrethroid tolerance.

The developmental diversity of P450 gene expression patterns is a very common phenomenon in insects [49,50], and tissue-specific expression patterns of detoxification genes are closely associated with their biological and physiological functions in insects [51,52]. In *S. frugiperda*, *CYP9A58* and *CYP9A59* exhibited similar expression patterns, with low transcript levels from the second- to fifth-instar larvae and peak expression in the sixth-instar larvae, and both P450 genes also showed elevated expression in the midgut. However, this pattern may reflect either a response to insecticide stress or developmental/physiological changes associated with feeding, gut microbiome dynamics, or metamorphosis. Additionally, *CYP9A58* and *CYP9A59* were highly expressed in the head of *S. frugiperda*, which may be associated with the detoxification of pyrethroid insecticides that target voltage-gated sodium channels (VGSCs). Similarly, the brain-specific expression of *CYP6BQ9* was involved in the detoxification of deltamethrin in *Tribolium castaneum* [53].

To functionally validate the role of these *P450* genes in pyrethroid detoxification in *S. frugiperda*, RNAi, as a powerful tool for studying the function of target genes in insects, was employed. For example, RNAi-mediated knockdown of *CYP321A7* and *CYP6AE43* significantly increased larval mortality rates in *S. frugiperda* after exposure to indoxacarb [54]. Similarly, silencing *CYP321A* genes in *S. litura* enhanced larval susceptibility to xenobiotics, including lambda-cyhalothrin [55]. In the present study, knockdown of *CYP9A58* or *CYP9A59* individually in *S. frugiperda* significantly increased larval mortality following lambda-cyhalothrin treatment, whereas no significant effect was observed for bifenthrin. We propose that, despite both pyrethroids sharing the same molecular target, structural differences, particularly the chiral configuration of bifenthrin, may influence its binding conformation with P450 enzymes, thereby affecting metabolic efficiency. Even though these genes are transcriptionally induced, the encoded enzymes may not serve as the primary metabolic pathway for bifenthrin [56,57]. This hypothesis is supported by molecular docking results, which indicate that CYP9A58 and CYP9A59 exhibit differential substrate preferences and catalytic efficiencies toward the two pyrethroids. Furthermore, bifenthrin metabolism likely involves multiple functionally redundant P450s; silencing one or even both of these genes may be compensated for by other P450 family members or alternative detoxification enzymes [58,59]. Consequently, the observed induction effects may represent a non-specific stress response rather than a dedicated detoxification mechanism.

Three-dimensional structural modeling and molecular docking serve as robust strategies to predict protein-substrate interactions, offering a mechanistic understanding of substrate selectivity and metabolic divergence [60]. Our results demonstrate that P450 proteins (CYP9A58 or CYP9A59) form stable complexes with pyrethroid insecticides, as evidenced by their negative binding free energies, indicating high binding affinity for bifenthrin or lambda-cyhalothrin. These findings are consistent with previous reports in *S. litura* and *S. frugiperda* [41,61]. Future studies should include additional validation using transgenic *Drosophila melanogaster* assays and other experimental approaches.

It is worth noting that, although this study demonstrates the metabolic detoxification roles of P450s *CYP9A58* and *CYP9A59* in laboratory-reared *S. frugiperda* against pyrethroid insecticides, metabolic detoxification mediated solely by overexpression of P450 genes is often insufficient to explain the extremely high levels of resistance observed in field populations. This strongly suggests that P450-mediated detoxification likely needs to act in concert with other resistance mechanisms, such as target-site mutations, reduced cuticular penetration, or behavioral avoidance, to reach the phenotypic thresholds observed in the field [62,63]. Therefore, the functional interpretation of P450s must be contextualized within the realistic backdrop of coexisting multi-mechanism resistance in the field. For a devastating agricultural pest like *S. frugiperda*, overlooking such synergistic effects may lead to a serious underestimation of the rate of resistance evolution in the field, thereby compromising the accuracy of resistance early-warning models. Future research should focus on dissecting the interaction networks between P450s and other potential mechanisms so as to provide a more robust theoretical foundation for precise management of field resistance.

## 5. Conclusions

Detoxification enzyme assays revealed that cytochrome P450 monooxygenases are likely involved in pyrethroid detoxification in *S. frugiperda*. Notably, P450 genes *CYP9A58* and *CYP9A59* were significantly upregulated by both bifenthrin and lambda-cyhalothrin, exhibiting distinct developmental stage- and tissue-specific expression patterns. Functional validation via RNAi, complemented by homology modeling and molecular docking analyses, confirmed that the upregulation of both *P450* genes plays a pivotal role in conferring tolerance to pyrethroid insecticides (including lambda-cyhalothrin) in *S. frugiperda*. These findings provide a theoretical framework for understanding the molecular mechanisms underlying P450-mediated pyrethroid detoxification, offering valuable insights for developing resistance management strategies against this pest.

## Figures and Tables

**Table 1:** Primers used for determining the expression level of P450 genes.

Name	Primer Sequence (5′-3′)	GenBank Accession Number
CYP9A58-QF	GCGGCAACTAACTTCAATT	KJ671577
CYP9A58-QR	CGTTCCTTCATCGTATCCA	
CYP9A59-QF	AGGTGAAGAGTGGAAGGA	MZ945705
CYP9A59-QR	TGGTTGTTCTCATCATTGTG	
CYP9A60-QF	CAAGGTCAAGAATGGAAGGA	MZ945685
CYP9A60-QR	CAGGAGGCAATCACATCAT	
EF-1α-QF	GACAAACGTACCATCGAGAAG	XM_035588389
EF-1α-QR	GATACCAGCCTCGAACTCAC	
α-TUB-QF	AGGGCTGTGTTTGTTGACT	XM_035600854
α-TUB-QR	TCCTTACCGATGGTGTAGTG	
RPL10-QF	TGGGTAAGAAGAAGGCTACG	XM_035580073
RPL10-QF	TGTTGATGCGGATGACAT	
SOD-QF	TCGGCACAATCATCAGTC	XM_035587671
SOD-QR	AGTCCTTCTCAATAGCCTGC	

**Table 2:** Primers used for RNA interference of P450 genes.

Name	Primer Sequence (5′-3′)
dsCYP9A58-F	taatacgactcactataGAAGTCCAGCGTTCACAAG
dsCYP9A58-R	taatacgactcactataGTCTCGTTCCTTCATCGTATC
dsCYP9A59-F	taatacgactcactataGTGCGACTGTTGAAGAATCT
dsCYP9A59-R	taatacgactcactataGGTATCCATAATTCTGTACCTT
dsGFP-F	taatacgactcactatagggCACAAGTTCAGCGTGTCCG
dsGFP-R	taatacgactcactatagggTTCACCTTGATGCCGTTC

T7 polymerase promoter sequences were represented by lowercase letters.

**Table 3:** Sensitivity of *S. frugiperda* to bifenthrin and lambda-cyhalothrin.

Insecticides	Slop ± SE	LC_25_ μg/mL (95% CI)	LC_50_ μg/mL (95% CI)	χ^2^ (df)	*p*-Value
bifenthrin	2.028 ± 0.268	58.851 (42.144–76.422)	126.585 (98.229–169.422)	9.253 (16)	0.903
lambda-cyhalothrin	1.614 ± 0.226	33.828 (21.485–46.714)	88.562 (65.806–122.353)	8.512 (16)	0.932

## Data Availability

The data presented in this study are included within the article. Further inquiries should be directed to the corresponding author.

## Supplementary Materials

The following supporting information can be downloaded at https://www.mdpi.com/article/10.3390/insects17080836/s1, Table S1: Raw data of the relative expression levels of target genes following exposure to pyrethroid insecticides; Table S2: Raw data of the spatiotemporal expression of target genes. Table S3: Raw data of the relative expression levels of target genes following dsRNA injection.

## Abbreviations

The following abbreviations are used in this manuscript:

CarE	Carboxylesterase
CDNB	1-Chloro-2,4-dinitrobenzene
7-EC	7-Ethoxycoumarin
FAW	Fall armyworm
GFP	Green fluorescent protein
GSH	Glutathione
GST	Glutathione S-transferase
P450	Cytochrome P450 monooxygenase
PBO	Piperonyl butoxide
qRT-PCR	Quantitative real-time polymerase chain reaction
RNAi	RNA interference
